# Supplementary material for: Rapid Development of Modified Vaccinia Virus Ankara (MVA)-Based Vaccine Candidates Against Marburg Virus Suitable for Clinical Use in Humans
Source: Vaccines (Basel). 2024 Nov 24;12(12):1316. doi: 10.3390/vaccines12121316 (PMC11680136; doi:10.3390/vaccines12121316)
Supplement: Supplementary file 1 [file vaccines-12-01316-s001.zip › vaccines-3291505-supplementary.pdf]

Supplementary Table S1: Antibodies for western blotting (WB) and immunofluorescence (IF)

Antibody	Company	Catalogue No.	Method	Dilution
GFP antibody [GT859] (HRP)	GeneTex	GTX628528-01	WB	1:1,000
mCherry Polyclonal Antibody	Invitrogen	PA5-34974	WB	1:1,000
MARV-GP antibody (clone 50-6-10)	BioGenes GmbH	n/a	WB	1:1,000
Anti-Marburg Virus Glycoprotein	IBT Bioservices	0303-007	IF	1:1,000
Anti-Marburg Virus Nucleoprotein [MARV 59-9-10-7]	Absolute Antibody	Ab01144-23.0	WB, IF	1:1,000
Anti-GAPDH	Thermo Fisher Scientific	MA5-15738	WB	1:2,000
Polyclonal goat anti-mouse immunoglobulins HRP	Agilent Technologies	P0447	WB	1:5,000
Anti-rabbit IgG HRP-linked anti-body	Cell Signaling Technol-ogy	7074S	WB	1:5,000
Goat anti-Mouse IgG (H+L) Highly Cross-Ad-sorbed Secondary Antibody, Alexa Fluor™ 568	Invitrogen	A11031	IF	1:1,000
Goat anti-Rabbit IgG (H+L) Highly Cross-Ad-sorbed Secondary Anti-body, Alexa Fluor™ 488	Invitrogen	A11034	IF	1:1,000

Supplementary Table S2: Predicted peptides for the stimulation of MARV-GP- and MARV-NP-specific T cells. n/a = not applicable

Name	Sequence	MHC Allele	Peptide pool	Overlapping peptide containing peptide
$GP_{184-191}$	FSLINRHAI	H2-Db	n/a	n/a
$GP_{490-498}$	LTLSYFPKI	H2-Kb	n/a	n/a
$GP_{583-591}$	FSLINRHAI	H2-Db	n/a	n/a
GP_{28-36}	IQPQNVDSV	H2-Db	GP Pool 1	n/a
GP_{47-54}	VHLMGFTL	H2-Kb	GP Pool 1	n/a
$GP_{332-341}$	SMPPHNTTTI	H2-Db	GP Pool 1	n/a
$GP_{333-341}$	MPPHNTTTI	H2-Db	GP Pool 1	n/a
$GP_{377-385}$	SAPSKTTLL	H2-Db	GP Pool 1	n/a
$GP_{547-555}$	GLIKNQNNL	H2-Db	GP Pool 1	n/a
$GP_{453-461}$	NAPIDFDPV	H2-Db	GP Pool 1	n/a
$GP_{142-149}$	AFFLYDRI	H2-Kb	GP Pool 2	n/a
$GP_{160-168}$	FTEGNIAAM	H2-Db	GP Pool 2	n/a
$GP_{429-437}$	VYFRRKRNI	H2-Kb	GP Pool 2	n/a
$GP_{453-461}$	NAPIDFDPV	H2-Db	GP Pool 2	n/a
$GP_{488-495}$	ISLTLSYF	H2-Kb	GP Pool 2	n/a
$GP_{488-498}$	ISLTLSYFPKI	H2-Kb	GP Pool 2	n/a
$GP_{531-540}$	SWIPFFGPGI	H2-Kb	GP Pool 2	n/a
$GP_{582-591}$	TFSLINRHAI	H2-Db	GP Pool 2	n/a
NP_{32-42}	VSICNQIIDAI	H2-Db	n/a	NP P8
$NP_{218-226}$	VGQTRFSGL	H2-Db	n/a	NP P54, NP P55

$NP_{463-473}$	FALLNEDEDTL	H2-Db	n/a	NP P116
$NP_{167-174}$	VTYPNHWL	H2-Db	NP Pool A1	NP P41, NP P42
$NP_{188-196}$	SSFILKFVL	H2-Db	NP Pool A1	NP P47
$NP_{285-294}$	SGINNLEHGL	H2-Kb	NP Pool A1	NP P71, NP P72
$NP_{452-459}$	SSSSFVDL	H2-Db	NP Pool A1	NP P112, NP P113
$NP_{642-650}$	RTFLYPNDL	H2-Kb	NP Pool A1	NP P160, NP P161
$NP_{102-112}$	VIPNEPHYSPL	H2-Db	NP Pool A2	NP P26
$NP_{131-138}$	LFLSFCSL	H2-Kb	NP Pool A2	NP P32, NP P33
$NP_{133-140}$	LSFCSLFL	H2-Kb	NP Pool A2	NP P33, NP P34
$NP_{133-143}$	LSFCSLFLPKL	H2-Kb	NP Pool A2	NP P33, NP P34
$NP_{188-197}$	SSFILKFVLI	H2-Db	NP Pool A2	NP P47
$NP_{260-269}$	TSFKQALSNL	H2-Db	NP Pool A2	NP P65

Supplementary Table S3: MARV-NP overlapping peptides

Name	Sequence	Pools
NP P1	MDLHSLLELGTKPTA	V1/H1
NP P2	SLLELGTKPTAPHVR	V1/H1
NP P3	LGTKPTAPHVRNKKV	V2/H1
NP P4	PTAPHVRNKKVILFD	V2/H1
NP P5	HVRNKKVILFDTNHQ	V3/H1
NP P6	KKVILFDTNHQVSIC	V3/H1
NP P7	LFDTNHQVSICNQII	V4/H1
NP P8	NHQVSICNQIIDAIN	V4/H1
NP P9	SICNQIIDAINSGID	V5/H1
NP P10	QIIDAINSGIDLGDL	V5/H1
NP P11	AINSGIDLGDLLEGG	V6/H1
NP P12	GIDLGDLLEGGLLTL	V6/H1
NP P13	GDLLEGGLLTLCVEH	V7/H1
NP P14	EGGLLTLCVEHYYNS	V7/H1
NP P15	LTLCVEHYYNSDKDK	V8/H1
NP P16	VEHYYNSDKDKFNTS	V8/H1
NP P17	YNSDKDKFNTSPIAR	V9/H1
NP P18	KDKFNTSPIARYLRD	V9/H1
NP P19	NTSPIARYLRDAGYE	V10/H1
NP P20	IARYLRDAGYEFDVI	V10/H1
NP P21	LRDAGYEFDVIKNAD	V11/H1
NP P22	GYEFDVIKNADATRF	V11/H1
NP P23	DVIKNADATRFLDVI	V1/H2
NP P24	NADATRFLDVIPNEP	V1/H2
NP P25	TRFLDVIPNEPHYSP	V2/H2
NP P26	DVIPNEPHYSPLILA	V2/H2
NP P27	NEPHYSPLILALKTL	V3/H2
NP P28	YSPLILALKTLESTE	V3/H2
NP P29	ILALKTLESTESQRG	V4/H2
NP P30	KTLESTESQRGRIGL	V4/H2
NP P31	STESQRGRIGLFLSF	V5/H2

NP P32	QRGRIGLFLSFCSLF	V5/H2
NP P33	IGLFLSFCSLFLPKL	V6/H2
NP P34	LSFCSLFLPKLVVGD	V6/H2
NP P35	SLFLPKLVVGDRASI	V7/H2
NP P36	PKLVVGDRASIEKAL	V7/H2
NP P37	VGDRASIEKALRQVT	V8/H2
NP P38	ASIEKALRQVTVHQE	V8/H2
NP P39	KALRQVTVHQEQGIV	V9/H2
NP P40	QVTVHQEQGIVTYPN	V9/H2
NP P41	HQEQGIVTYPNHWLT	V10/H2
NP P42	GIVTYPNHWLTTGHM	V10/H2
NP P43	YPNHWLTTGHMKVIF	V11/H2
NP P44	WLTTGHMKVIFGILR	V11/H2
NP P45	GHMKVIFGILRSSFI	V1/H3
NP P46	VIFGILRSSFILKFV	V1/H3
NP P47	ILRSSFILKFVLIHQ	V2/H3
NP P48	SFILKFVLIHQGVNL	V2/H3
NP P49	KFVLIHQGVNLVTGH	V1/H6
NP P50	IHQGVNLVTGHDAYD	V3/H3
NP P51	VNLVTGHDAYDSIIS	V4/H3
NP P52	TGHDAYDSIISNSVG	V4/H3
NP P53	AYDSIISNSVGQTRF	V5/H3
NP P54	IISNSVGQTRFSGLL	V5/H3
NP P55	SVGQTRFSGLLIVKT	V6/H3
NP P56	TRFSGLLIVKTVLEF	V6/H3
NP P57	GLLIVKTVLEFILQK	V7/H3
NP P58	VKTVLEFILQKTDSG	V7/H3
NP P59	LEFILQKTDSGVTLH	V8/H3
NP P60	LQKTDSGVTLHPLVR	V8/H3
NP P61	DSGVTLHPLVRTSKV	V9/H3
NP P62	TLHPLVRTSKVKNEV	V9/H3
NP P63	LVRTSKVKNEVTSFK	V10/H3
NP P64	SKVKNEVTSFKQALS	V10/H3
NP P65	NEVTSFKQALSNLAR	V11/H3
NP P66	SFKQALSNLARHGEY	V11/H3
NP P67	ALSNLARHGEYAPFA	V1/H4
NP P68	LARHGEYAPFARVLN	V1/H4
NP P69	GEYAPFARVLNLSGI	V2/H4
NP P70	PFARVLNLSGINNLE	V2/H4
NP P71	VLNLSGINNLEHGLY	V3/H4
NP P72	SGINNLEHGLYPQLS	V3/H4
NP P73	NLEHGLYPQLSAIAL	V4/H4
NP P74	GLYPQLSAIALGVAT	V4/H4
NP P75	QLSAIALGVATAHGS	V5/H4
NP P76	IALGVATAHGSTLAG	V5/H4

NP P77	VATAHGSTLAGVNVG	V6/H4
NP P78	HGSTLAGVNVGEQYQ	V6/H4
NP P79	LAGVNVGEQYQQLRE	V7/H4
NP P80	NVGEQYQQLREAAHD	V7/H4
NP P81	QYQQLREAAHDAEVK	V8/H4
NP P82	LREAAHDAEVKLQRR	V8/H4
NP P83	AHDAEVKLQRRHERQ	V9/H4
NP P84	EVKLQRRHERQEIQA	V9/H4
NP P85	QRRHERQEIQAIAED	V10/H4
NP P86	ERQEIQAIAEDDEER	V10/H4
NP P87	IQAIAEDDEERKILE	V11/H4
NP P88	AEDDEERKILEQFHL	V11/H4
NP P89	EERKILEQFHLQKTE	V1/H5
NP P90	ILEQFHLQKTEITHS	V1/H5
NP P91	FHLQKTEITHSQTLA	V2/H5
NP P92	KTEITHSQTLAVLSQ	V2/H5
NP P93	THSQTLAVLSQKREK	V3/H5
NP P94	TLAVLSQKREKLARL	V3/H5
NP P95	LSQKREKLARLAAEI	V4/H5
NP P96	REKLARLAAEIENNI	V4/H5
NP P97	ARLAAEIENNIVEDQ	V5/H5
NP P98	AEIENNIVEDQGFKQ	V5/H5
NP P99	NNIVEDQGFKQSQNR	V6/H5
NP P100	EDQGFKQSQNRVSQS	V6/H5
NP P101	FKQSQNRVSQSFLND	V7/H5
NP P102	QNRVSQSFLNDPTPV	V7/H5
NP P103	SQSFLNDPTPVEVTV	V8/H5
NP P104	LNDPTPVEVTVQARP	V8/H5
NP P105	TPVEVTVQARPINRP	V9/H5
NP P106	VTVQARPINRPTALP	V9/H5
NP P107	ARPINRPTALPPPVD	V10/H5
NP P108	NRPTALPPPVDGKIE	V10/H5
NP P109	ALPPPVDGKIEHEST	V11/H5
NP P110	PVDGKIEHESTEDSS	V11/H5
NP P111	KIEHESTEDSSSSSS	V1/H6
NP P112	ESTEDSSSSSSFVDL	V2/H6
NP P113	DSSSSSSFVDLNDPF	V3/H6
NP P114	SSSFVDLNDPFALLN	V4/H6
NP P115	VDLNDPFALLNEDED	V5/H6
NP P116	DPFALLNEDEDTLDD	V6/H6
NP P117	LLNEDEDTLDDSVMI	V7/H6
NP P118	DEDTLDDSVMIPSTT	V8/H6
NP P119	LDDSVMIPSTTSREF	V9/H6
NP P120	VMIPSTTSREFQGIS	V10/H6
NP P121	STTSREFQGISEPPR	V11/H6

NP P122	REFQGISEPPRQSQD	V1/H7
NP P123	GISEPPRQSQDLDNS	V2/H7
NP P124	PPRQSQDLDNSQGKQ	V3/H7
NP P125	SQDLDNSQGKQEDES	V4/H7
NP P126	DNSQGKQEDESTNLI	V5/H7
NP P127	GKQEDESTNLIKKPF	V1/H7
NP P128	DESTNLIKKPFLRYQ	V2/H7
NP P129	NLIKKPFLRYQELPP	V3/H7
NP P130	KPFLRYQELPPVQED	V4/H7
NP P131	RYQELPPVQEDDESE	V5/H7
NP P132	LPPVQEDDESEYTTD	V6/H7
NP P133	QEDDESEYTTDSQES	V7/H7
NP P134	ESEYTTDSQESIDQP	V8/H7
NP P135	TTDSQESIDQPGSDN	V9/H7
NP P136	QESIDQPGSDNEQGV	V10/H7
NP P137	DQPGSDNEQGVDLPP	V11/H7
NP P138	SDNEQGVDLPPPPLY	V1/H8
NP P139	QGVDLPPPPLYAQEK	V2/H8
NP P140	LPPPPLYAQEKRQDP	V3/H8
NP P141	PLYAQEKRQDPIQHP	V4/H8
NP P142	QEKRQDPIQHPAVSS	V5/H8
NP P143	QDPIQHPAVSSQDPF	V6/H8
NP P144	QHPAVSSQDPFGSIG	V7/H8
NP P145	VSSQDPFGSIGDVNG	V8/H8
NP P146	DPFGSIGDVNGDILE	V9/H8
NP P147	SIGDVNGDILEPIRS	V10/H8
NP P148	VNGDILEPIRSPSSP	V11/H8
NP P149	ILEPIRSPSSPSAPQ	V1/H9
NP P150	IRSPSSPSAPQEDTR	V2/H9
NP P151	SSPSAPQEDTRAREV	V3/H9
NP P152	APQEDTRAREVYELS	V4/H9
NP P153	DTRAREVYELSPDFT	V5/H9
NP P154	REVYELSPDFTNYED	V6/H9
NP P155	ELSPDFTNYEDDQQN	V7/H9
NP P156	DFTNYEDDQQNWPQR	V8/H9
NP P157	YEDDQQNWPQRVVTK	V9/H9
NP P158	QQNWPQRVVTKKGRT	V10/H9
NP P159	PQRVVTKKGRTFLYP	V11/H9
NP P160	VTKKGRTFLYPNDLL	V1/H10
NP P161	GRTFLYPNDLLQTNP	V2/H10
NP P162	LYPNDLLQTNPPESL	V3/H10
NP P163	DLLQTNPPESLMTAL	V4/H10
NP P164	TNPPESLMTALVEEY	V5/H10
NP P165	ESLMTALVEEYQNPV	V6/H10
NP P166	TALVEEYQNPVSAKE	V7/H10

NP P167	EEYQNPVSAKELQAD	V8/H10
NP P168	NPVSAKELQADWPDM	V9/H10
NP P169	AKELQADWPDMSFDE	V10/H10
NP P170	QADWPDMSFDERRHV	V11/H10
NP P171	PDMSFDERRHVAMNL	V11/H10

Supplementary Table S4: Antibodies and staining reagents for flow cytometry

Antigen	Clone	Fluorophore	Company	Catalogue No.
CD3	17A2	PE-Cy7	BioLegend	503832
CD4	GK1.5	Brilliant Violet 421	BioLegend	100437
CD8α	53-6.8	Alexa Fluor 488	BioLegend	100723
CD16/CD32	93	N/A	BioLegend	101320
IFN-γ	XMG1.2	APC	BioLegend	505810
TNF-α	MP6-XT22	PE	BioLegend	506306

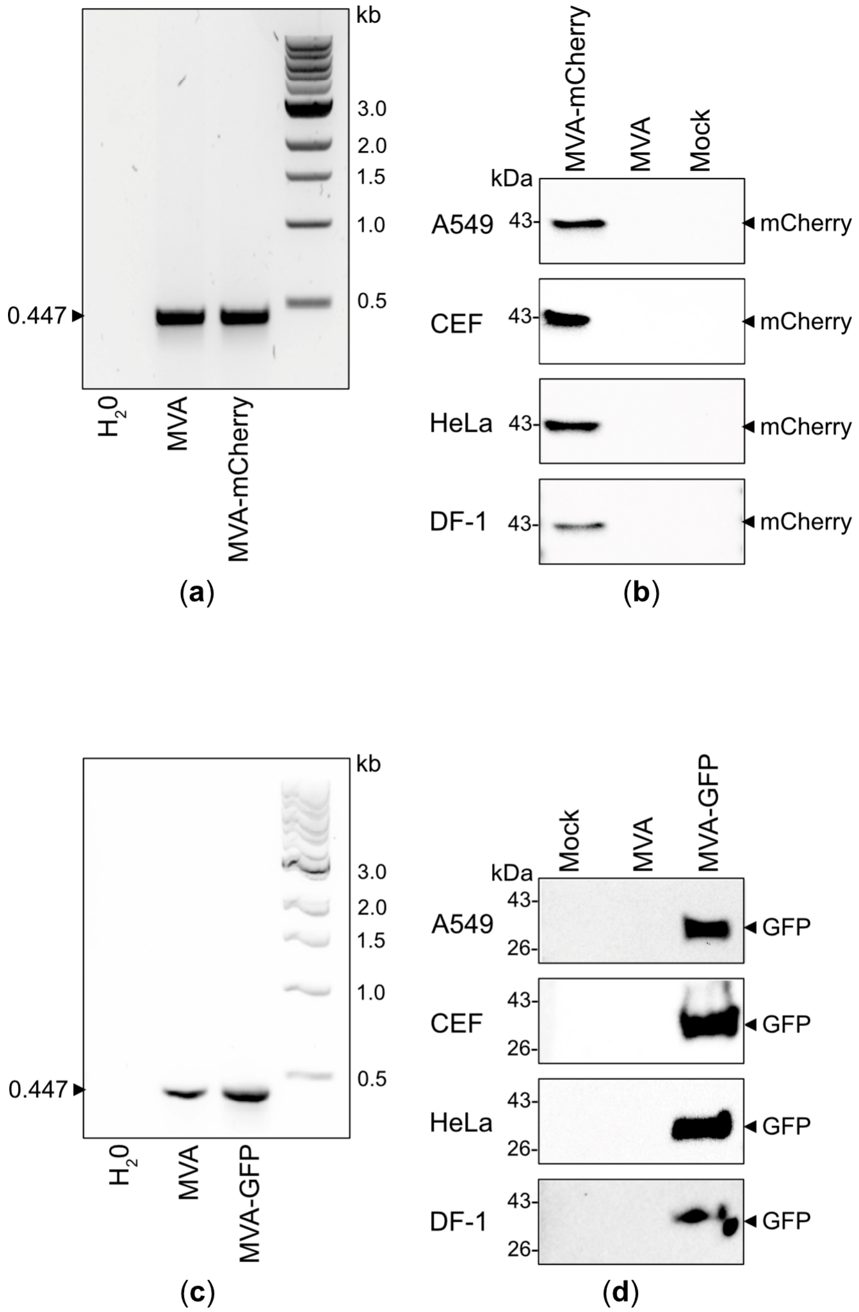

Figure S1. PCR analysis of viral DNA to monitor **(a, c)** the C7L gene locus in the MVA-mCherrysf (MVA-mCherry) and MVA-p11GFPsf (MVA-GFP) genome. Amplification of a specific 0.447 kb DNA fragment from the MVA-C7L gene sequence confirmed integrity of the C7L gene locus within the MVA genome. The MVA-C7L gene function is not essential for growth in chicken fibroblast cultures but is necessary to maintain unimpaired expression of MVA and recombinant genes that are under transcriptional control of vaccinia virus-specific late promoters [1]. **(b, d)** Synthesis of mCherry and GFP in MVA-GFP or MVA-mCherry infected cells. Cells were infected with MVA-mCherry or MVA-GFP at a MOI of 5 and collected 24 hours post infection (hpi). Polypeptides in cell lysates were separated by SDS-PAGE and analyzed with a monoclonal antibody targeting mCherry (1:5000) or GFP (1:5000). Lysates from non-infected cells (Mock) and cells infected with non-recombinant MVA (MVA) served as controls.

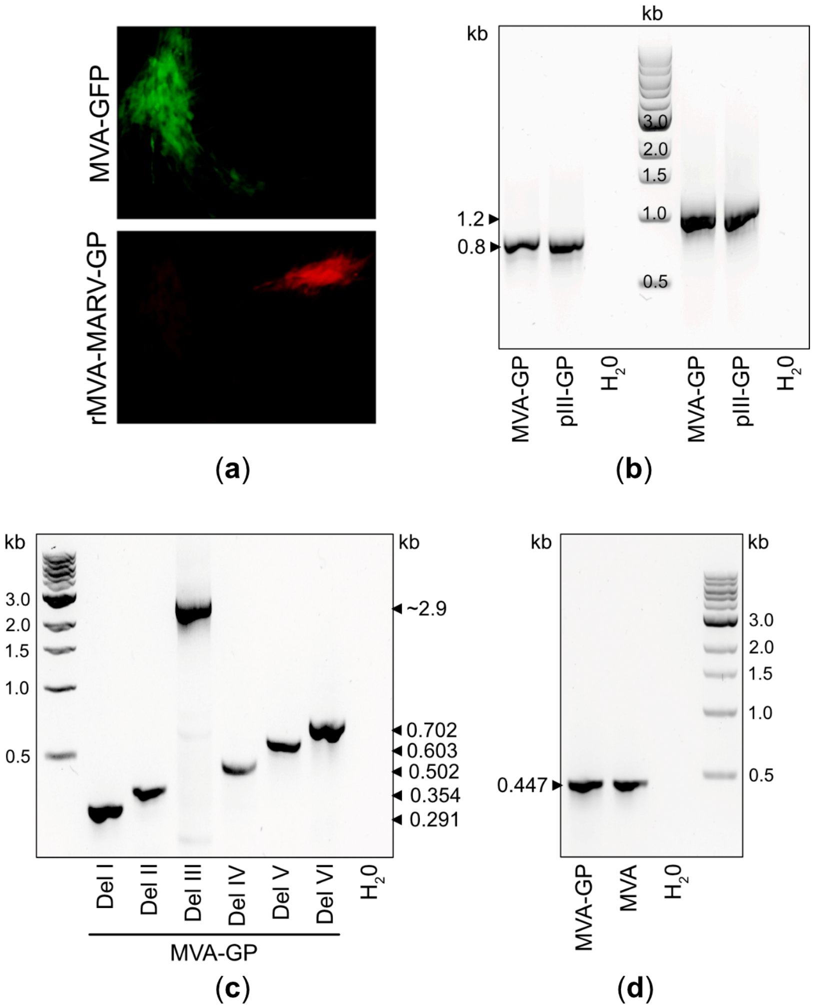

Figure S2. (a) Fluorescent microscopy of CEF cells to distinguish plaques from non-recombinant backbone virus MVA-GFP (green) and recombinant MVA-MARV-GP (red). **(b-d)** PCR analysis of viral DNA to monitor **(b)** the MARV-GP gene, **(c)** the six major deletion sites I to VI and **(d)** the C7L gene locus in the MVA-MARV-GP (MVA-GP) genome [1]. **(b)** Two different PCRs served to assess the integrity of the full-length MARV-GP gene sequence inserted in the MVA-GP genome. Amplified DNA fragments demonstrated the expected molecular weights with 1.2 kb (specific for GP gene nucleotides 761-1936) and 0.8 kb (nucleotides 454-1262). **(c)** PCR analysis of genomic viral DNA demonstrated the genetic stability for six loci in the MVA-MARV-GP genome (deletion sites Del I-VI) including the heterologous MARV-GP gene sequence inserted into the site of deletion III (Del III) with the amplification of characteristic size DNA fragments. **(d)** Amplification of a specific 0.447 kb DNA fragment from the MVA-C7L gene sequence demonstrated integrity of the C7L gene locus in the MVA genome.

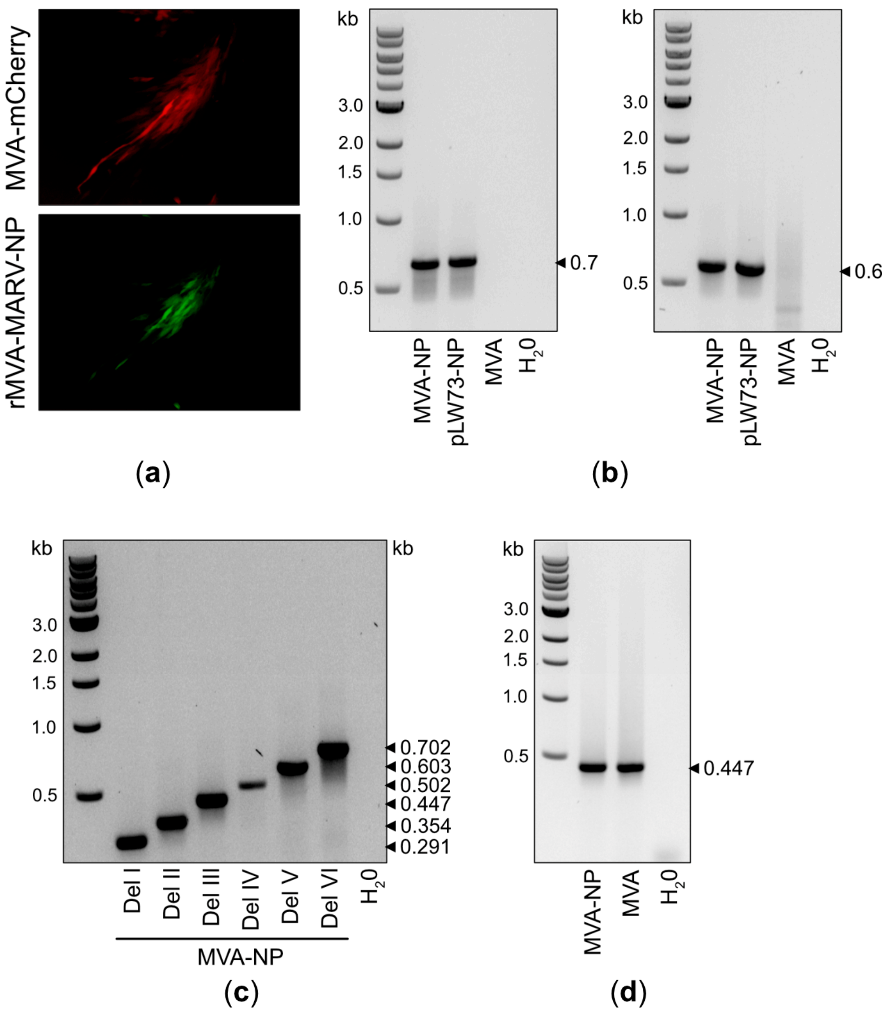

Figure S3. **(a)** Fluorescent microscopy of CEF cells to distinguish plaques from non-recombinant backbone virus MVA-mCherry (red) and recombinant MVA-MARV-NP (green). **(b-d)** PCR analysis of viral DNA to monitor **(b)** the MARV-NP gene, **(c)** the six major deletion sites I to VI, and **(d)** the C7L gene locus in the MVA-MARV-NP (MVA-NP) genome [1]. **(b)** Two different PCRs served to assess the integrity of the full-length MARV-NP gene sequence inserted in the MVA-GP genome. Amplified DNA fragments demonstrated the expected molecular weights with 0.7 kb (specific for NP gene nucleotides 413-1068) and 0.6 kb (nucleotides 1047-1694). **(c)** PCR analysis of genomic viral DNA demonstrated the genetic stability for six loci in the MVA-MARV-NP genome (deletion sites Del I-VI). **(d)** Amplification of a specific 0.447 kb DNA fragment from the MVA-C7L gene sequence demonstrated integrity of the C7L gene locus in the MVA genome.

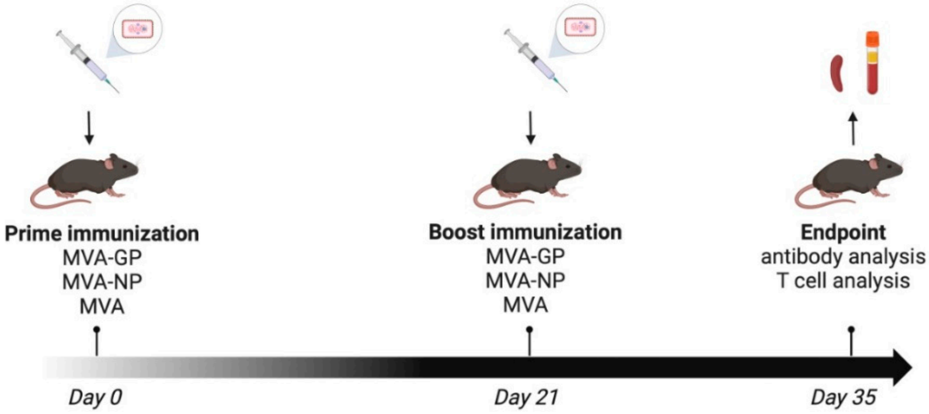

(a)

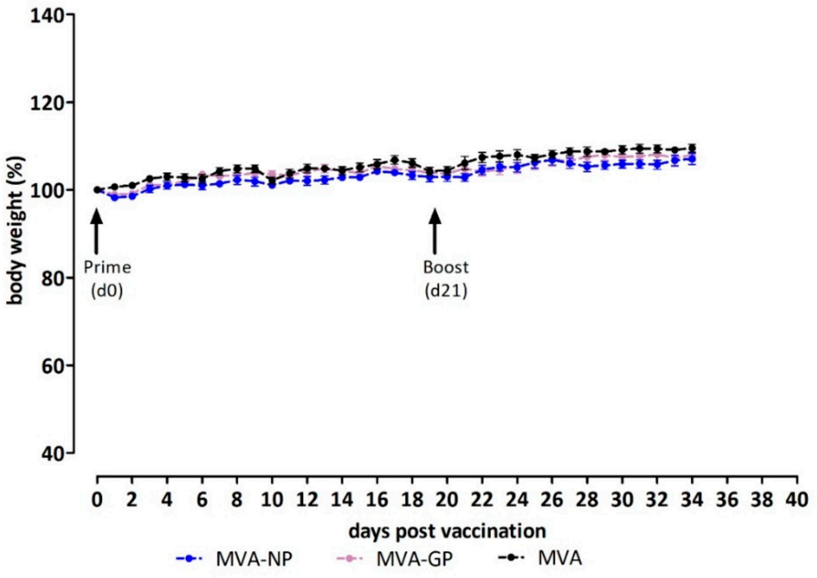

(b)

Figure S4. MVA-MARV-GP (MVA-GP) and MVA-MARV-NP (MVA-NP) immunization schedules and monitoring for side effects of vaccination. Groups of C57BL/6J mice (n=6-10) were immunized twice with 10^7 PFU via the intramuscular (i.m.) route over a 21-day interval. **(a)** Schematic diagram of the immunization schedule. T cell responses and antibody responses were tested at day 35 after prime immunization. **(b)** Monitoring for body weight changes of mice after prime-boost vaccination with MVA-GP and MVA-NP. Vaccination with non-recombinant MVA (MVA) served as control. Body weights were measured daily. No signs of side effects or disease were observed in immunized or control mice.

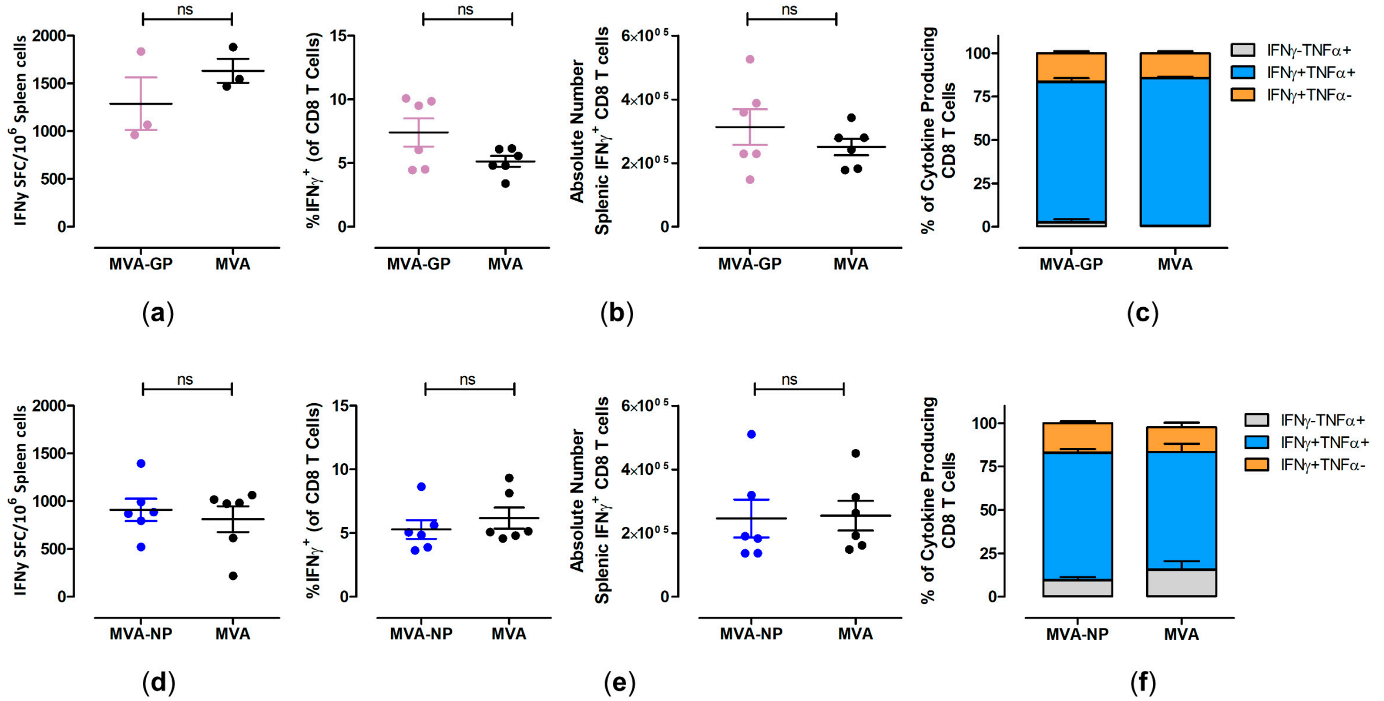

Figure S5. Activation of MVA-specific CD8+ T cells after prime-boost immunization with MVA-MARV-GP (MVA-GP) or MVA-MARV-NP (MVA-NP). Groups of C57BL/6J mice (n= 3-6) were i.m. immunized twice with 10^7 PFU MVA-GP or MVA-NP. Mice immunized with non-recombinant MVA served as controls. Splenocytes were collected and prepared on day 14 after boost immunization. Total splenocytes were stimulated with the H2d restricted MVA-specific peptide $B8R_{20-27}$ (TSYKFESV) measured by IFN-y ELISPOT assay and IFN-γ and TNF-α ICS plus FACS analysis. **(a, d)** IFN-y spot forming colonies (SFC) for stimulated splenocytes measured by ELISPOT assay. **(b, e)** IFN-**γ** production by CD8+ T cells measured by FACS analysis. Graphs show the frequency and absolute number of IFN-γ + CD8+ T cells. **(c, f)** Cytokine profile of MVA-specific CD8 T cells. Graphs show the mean frequency of IFN-γ-TNF-α+, IFN-γ+TNF-α+ and IFN-γ+TNF-α- cells within the cytokine positive CD8 T cell compartment. Error bars in scatter and bar graphs show the mean + standard error of the mean (SEM). Groups were analyzed by two-tailed Mann Whitney U test. ns = not significant.

References

1. Backes, S.; Sperling, K.M.; Zwilling, J.; Gasteiger, G.; Ludwig, H.; Kremmer, E.; Schwantes, A.; Staib, C.; Sutter, G. Viral host-range factor C7 or K1 is essential for modified vaccinia virus Ankara late gene expression in human and murine cells, irrespective of their capacity to inhibit protein kinase R-mediated phosphorylation of eukaryotic translation initiation factor 2alpha. *J. Gen. Virol.* **2010**, *91*, 470–482, doi:10.1099/vir.0.015347-0.

Original Picture western blotting and PCR

Figure 1d

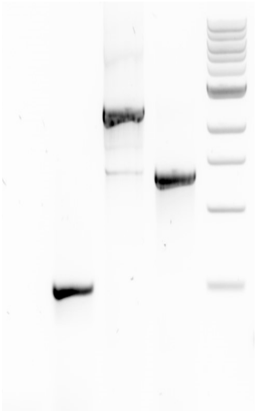

Figure 1f + Supplementary Figure S1c

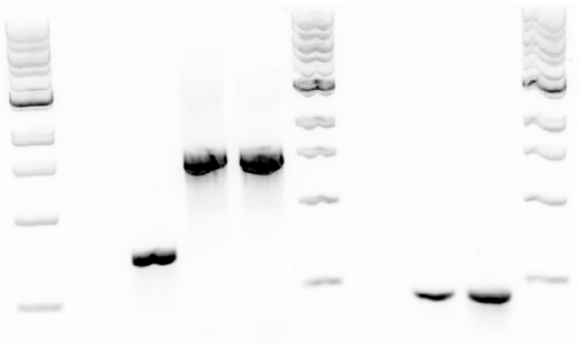

Figure 2b

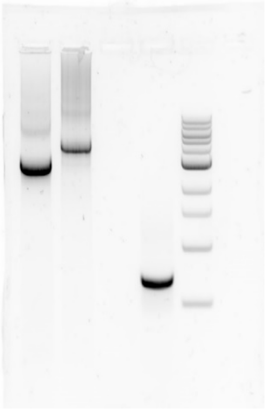

Figure 2e

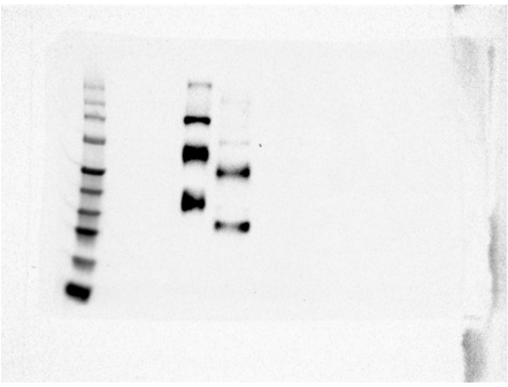

Figure 2f_MARV-GP

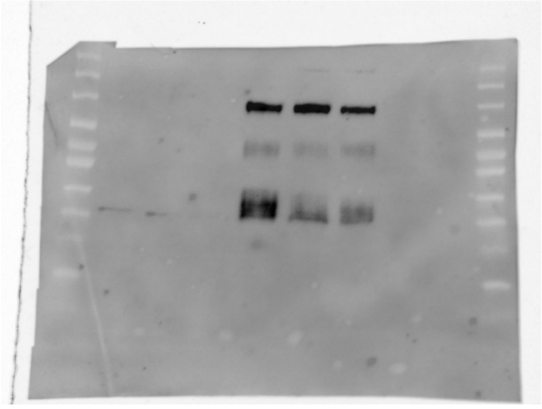

Figure 2f_GAPDH

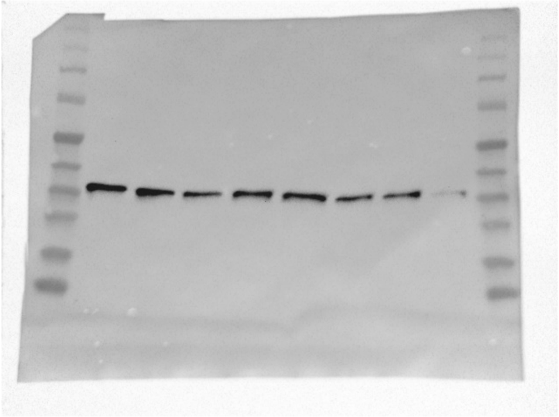

Figure 3b

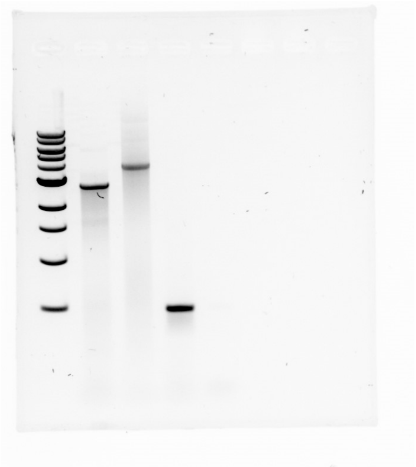

Figure 3e_MARV-NP

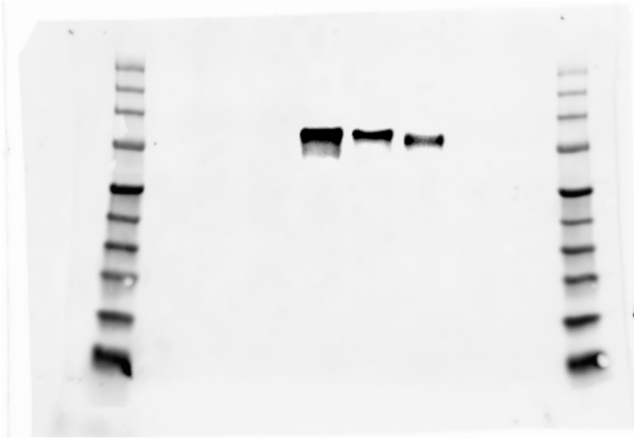

Figure 3e_GAPDH

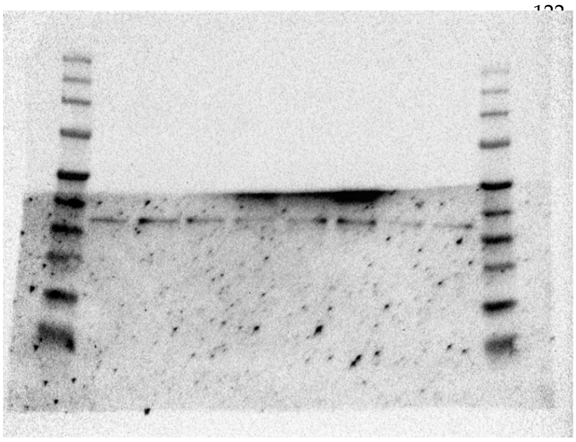

Supplementary Figure S1a

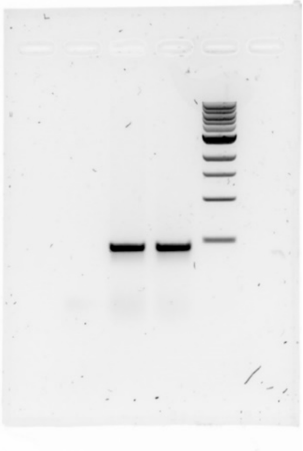

Supplementary Figure S1b_A549_ HeLa_DF-1

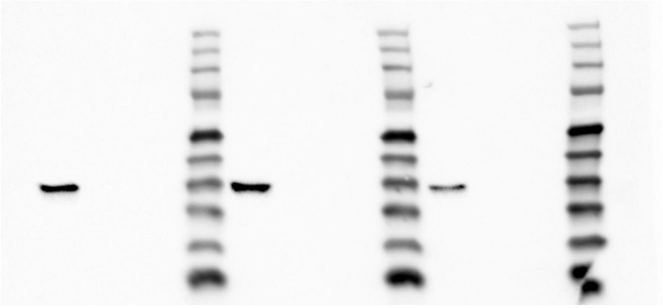

Supplementary Figure S1b_CEF_DF-1

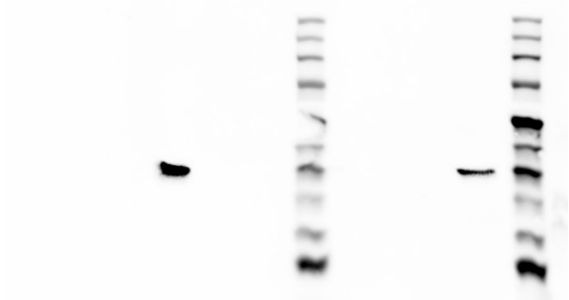

Supplementary Figure S1d_CEF_DF-1

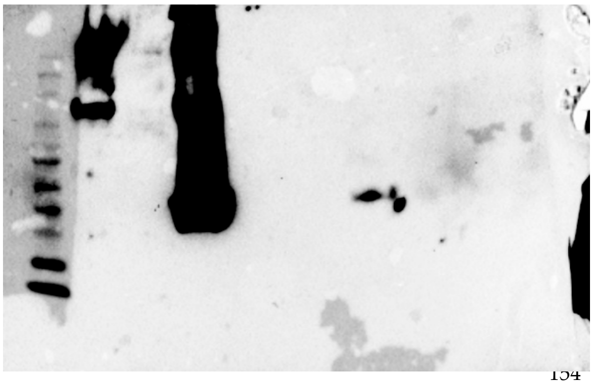

Supplementary Figure S1d_CEF_DF-1

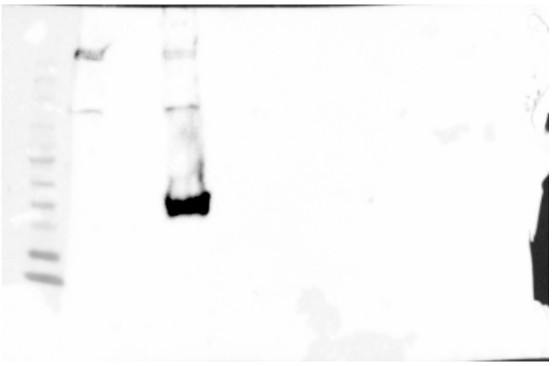

Supplementary Figure S1d_HeLa_A549

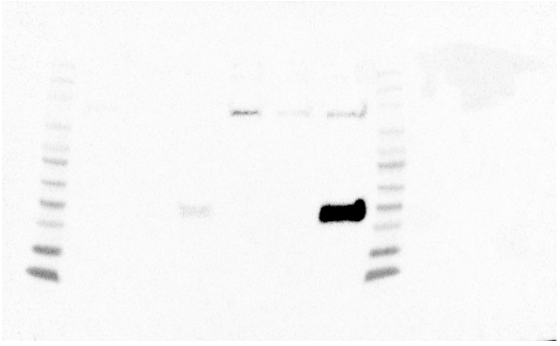

Supplementary Figure S1d_HeLa_A549

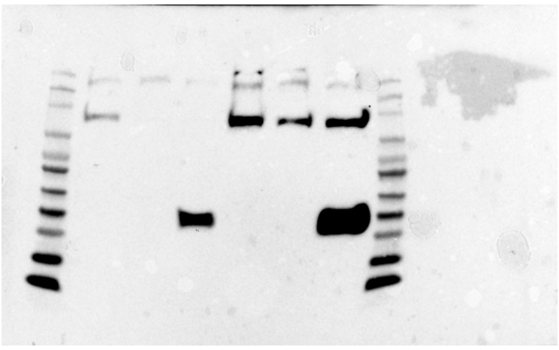

Supplementary Figure S2b

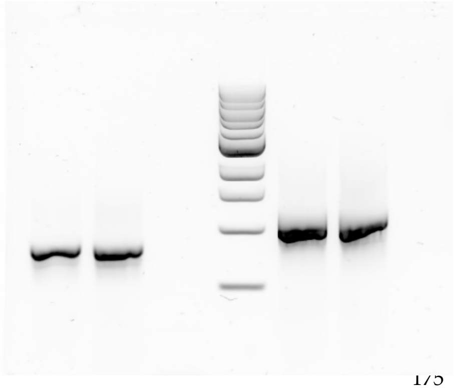

Supplementary Figure S2c

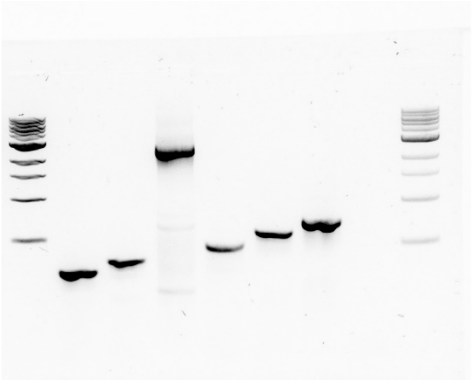

Supplementary Figure S2d

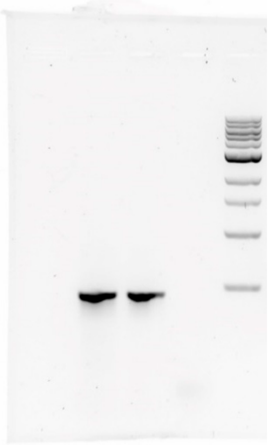

Supplementary Figure S3b

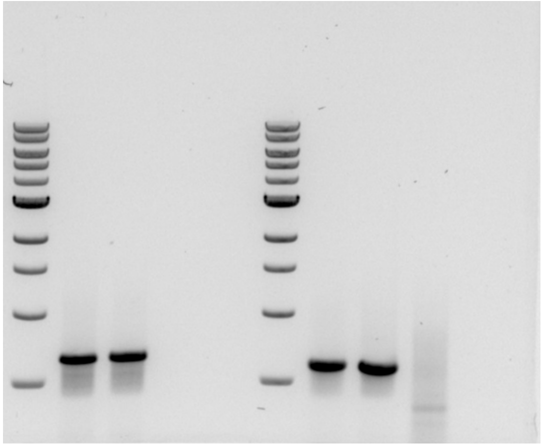

Supplementary Figure S3c

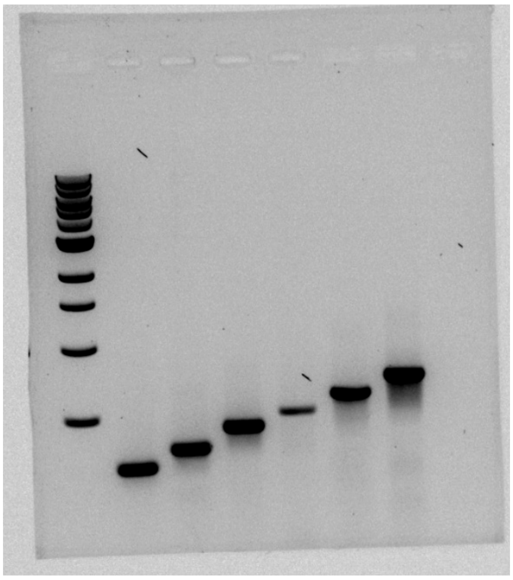

Supplementary Figure S3d

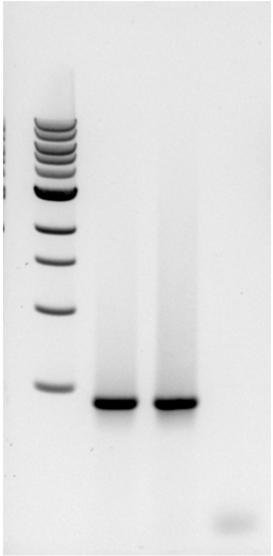

Figure 2e

Lane	Area
GP1/2	9878.711
preGP	20926.530
preGP	9671.652
GP2	21960.137
GP2	12295.075

Figure 2f (GP 1/2)

Lane	Area
Mock 0h	0
MVA 0h	0
MARV-GP 0h	0
MARV-GP 8h	1140.125
MARV-GP 24h	12270.296
MARV-GP 48h	8711.175
Mock 48h	0
MVA 48h	0

Figure 2f (GAPDH)

Lane	Area
Mock 0h	9172.054
MVA 0h	8717.589
MARV-GP 0h	5336.569
MARV-GP 8h	7847.711
MARV-GP 24h	9180.246
MARV-GP 48h	5263.933
Mock 48h	5122.397
MVA 48h	745.062

Figure 2f (GP2)

Lane	Area
Mock 0h	0
MVA 0h	0
MARV-GP 0h	0
MARV-GP 8h	26267.714
MARV-GP 24h	8635.773
MARV-GP 48h	7582.217
Mock 48h	0
MVA 48h	0

Figure 2f (Pre-GP)

Lane	Area
Mock 0h	0
MVA 0h	0
MARV-GP 0h	0
MARV-GP 8h	15262.622
MARV-GP 24h	7928.551
MARV-GP 48h	12679.693
Mock 48h	0
MVA 48h	0

Figure 3e (GAPDH)

Lane	Area
Mock 0h	2634.669
MVA 0h	4595.054
MARV-NP 0h	3226.983
MARV-NP 8h	1706.811
MARV-NP 24h	2187.033
MARV-NP 48h	3960.368
Mock 48h	1714.447
MVA 48h	2124.426

Figure 3e (NP)

Lane	Area
Mock 0h	0
MVA 0h	0
MARV-NP 0h	0
MARV-NP 8h	19073.267
MARV-NP 24h	10116.033
MARV-NP 48h	4337.861
Mock 48h	0
MVA 48h	0

Figure S1d

Lane	Area
A549_GFP	23327.116
CEF_GFP	43740.007
HeLa_GFP	65457.563
DF-1_GFP	31896.019
